# The green-brown polymorphism of the club-legged grasshopper *Gomphocerus sibiricus* is heritable and appears genetically simple

**DOI:** 10.1186/s12862-020-01630-7

**Published:** 2020-06-01

**Authors:** Holger Schielzeth, Petra Dieker

**Affiliations:** 1 Population Ecology Group, Institute of Ecology and Evolution, Friedrich Schiller University Jena, Dornburger Straße 159, 07743 Jena, Germany; 2 Present Address: Thünen Institute of Biodiversity, Bundesallee 65, 38116 Braunschweig, Germany

**Keywords:** Acrididae, Color polymorphism, Gomphocerinae, Heritability, Orthoptera, Mendelian inheritance, Phenocopies

## Abstract

**Background:**

Local coexistence of distinct, genetically determined color morphs can be unstable and transitional. Stable, long-term coexistence requires some form of balancing selection to protect morphs from getting lost by directional selection or genetic drift. However, not all phenotypic polymorphism need to have a genetic basis. We here report on the genetic basis of two color polymorphisms in the club-legged grasshopper *Gomphocerus sibiricus*: a green-brown polymorphism that is phylogenetically and geographically widespread among orthopteran insects and a pied-brown pattern polymorphism that is shared among many gomphocerine grasshoppers.

**Results:**

We found a remarkably clear outcome of matings within and between morph that suggest not only that the green-brown polymorphism is heritable in this species, but that results can be most parsimoniously explained by a single autosomal locus with two alleles in which the green allele is dominant over the brown allele. A few individuals did not match this pattern and suggest the existence of genetic modifiers and/or developmental phenocopies. We also show that the pied-brown polymorphism is highly heritable, although the evidence for the involvement of one or more loci is less clear-cut.

**Conclusions:**

Overall, our data demonstrate that the two polymorphisms are heritable in the club-legged grasshopper and appear genetically simple, at least with respect to green morphs. The results are consistent with the idea that the synthesis or transport of a pigment involved in the production of green coloration (likely biliverdin) is lost by homozygosity for loss-of-function alleles in brown individuals. The apparently simple genetic architecture of the green-brown polymorphism offer potential for studying balancing selection in the field and for genetic mapping in this species.

## Background

Color polymorphisms have fascinated evolutionary biologists for a long time, and indeed the analysis of color polymorphisms was the key observation in the discovery of inheritance [1–3]. The long-term coexistence of multiple discrete color variants (independent of obvious modifiers such as sex, age and condition) poses the question of how color polymorphisms are maintained. A narrow-sense definition of color polymorphisms is focused on phenotypic polymorphisms that have a genetic basis, with the rarest morph being too common to be explained by novel mutations [1, 4]. Genetic color polymorphisms offer superb natural systems for studying the maintenance of intraspecific variation by balancing selection [5]. However, polymorphisms in color are foremost a phenotypic feature of a population and not all phenotypic color polymorphisms need to have a genetic basis [6]. Both genetic and environmentally induced polymorphisms call for a more detailed understanding of how they are formed and maintained, but the evolutionary dynamics are very different depending on how color variation is transmitted across generations.

Orthopteran insects offer a fascinating system for the study of color polymorphisms [7, 8]. About 30% of all European orthopterans are green-brown polymorphic [9] and this encompasses species form the suborder Ensifera (e.g. *Decticus verrucivorus*) and the suborder Caelifera (e.g. *Pseudochorthippus parallelus*) that have diverged about 200 Mya [10]. Furthermore, some other orders of polyneopteran insects, like the praying mantises and stick insects [11] that have diverged from the Orthoptera about 250 Mya [10], show an equivalent green-brown polymorphism. The polymorphism is also geographically widespread across the world such that, for example, more than 45% of East African acridid grasshoppers are green-brown polymorphic [7]. It is therefore of key interest if this polymorphism in such a large and old clade of insects is homologous in its mechanistic underpinnings.

Orthopterans are also known for a phenomenon called homochromy, which describes the observation that local populations often match the predominant color of the local habitat [12]. Most of the reports on homochromy refer to other color polymorphisms, in particular to overall darkness and tone in Tetrigidae, Odipodinae and Acridinae (e.g. [7, 13–15]). But green-brown color morph ratios are also spatially variable and appear to depend on local environmental conditions [16, 17]. In an interspecific context there are patterns of habitat-dependence of the green-brown polymorphism, which is particularly common in species that inhabit grasslands [9]. This illustrates that body color is ecologically relevant in this group, probably because of their link to detectability and predator avoidance [18] and/or thermoregulation [19].

The evidence of genetic versus environmental control of the green-brown polymorphism is ambiguous in orthopterans. While there are several, mostly older, studies that have reported remarkable ontogenetic plasticity in species of the genera *Schistocerca*, *Acrida*, and *Oedaleus* [20–24] and also in the gomphocerine grasshopper *Syrbula admirabilis* [25], there is both older and newer evidence that the green-brown polymorphism is not under environmental, but rather under genetic control [26–28]. There thus seem to be clade- or species-specific differences in in genetic versus environmental determination and, because of the relevance to the maintenance of the polymorphism, more studies are needed to understand how the polymorphism is formed.

**Fig. 1:**
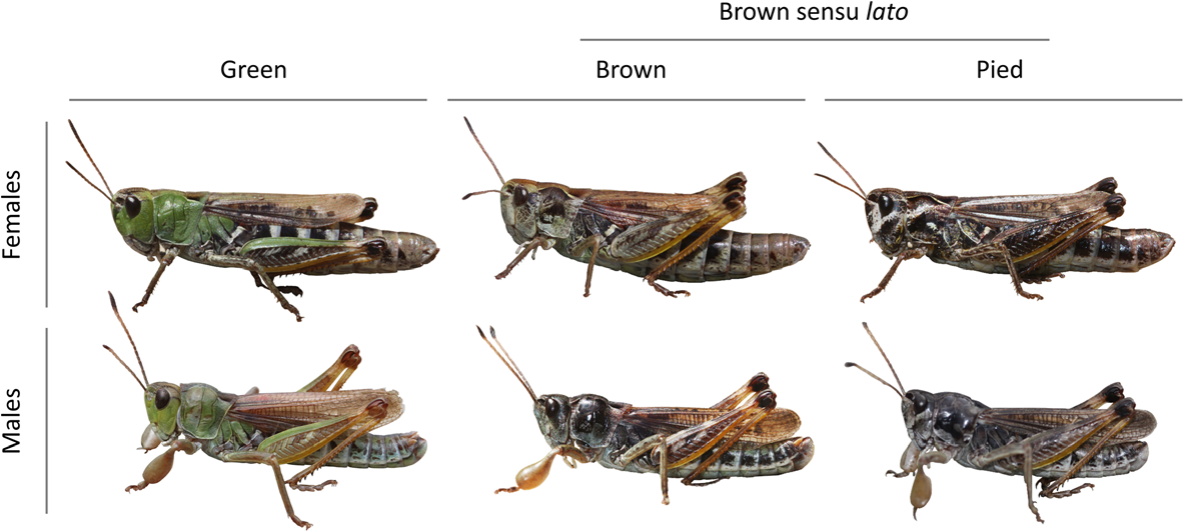
Color morphs in the club-legged grasshoppers *Gomphocerus sibiricus*. Green morphs are easily identified by the presence of green. Brown and pied morph both lack green and are therefore considered brown sensu *lato*. Pied morphs show a characteristic pale transverse line (bordered by dark areas) running from the sides of the head across the lateral lobes of the pronotum. Pied individual typically also show a dark patch on the frons, in particular in females

We set up a breeding experiment with the gomphocerine grasshopper *Gomphocerus sibiricus* (club-legged grasshopper, Caelifera: Acrididae) to elucidate the mode of color determination in this species (Fig. 1). We focus on the green-brown polymorphism that is more widely shared across orthopterans (and polyneopterans). Furthermore, we study the inheritance of a distinct variant of the brown morph that we call pied [16] and that is shared among many species within the large subfamily Gomphocerinae (hereinafter called the pied-brown polymorphism). Individuals were collected from the field and the mating design was established from virgin individuals in the laboratory. We had previously shown that club-legged grasshoppers do not switch color between green and brown morphs during ontogeny [29], but a formal proof of genetic inheritance has been lacking. We scored offspring color morphs and found a remarkably clear pattern of heritable color morph variation, both with respect to the green and the pied morph. Only very few green individuals did not match a simple inheritance pattern, suggesting that the basic inheritance pattern is largely genetically simple, with the possibility of additional genetic modifiers or occasional environment-induced phenocopies.

## Results

**Fig. 2:**
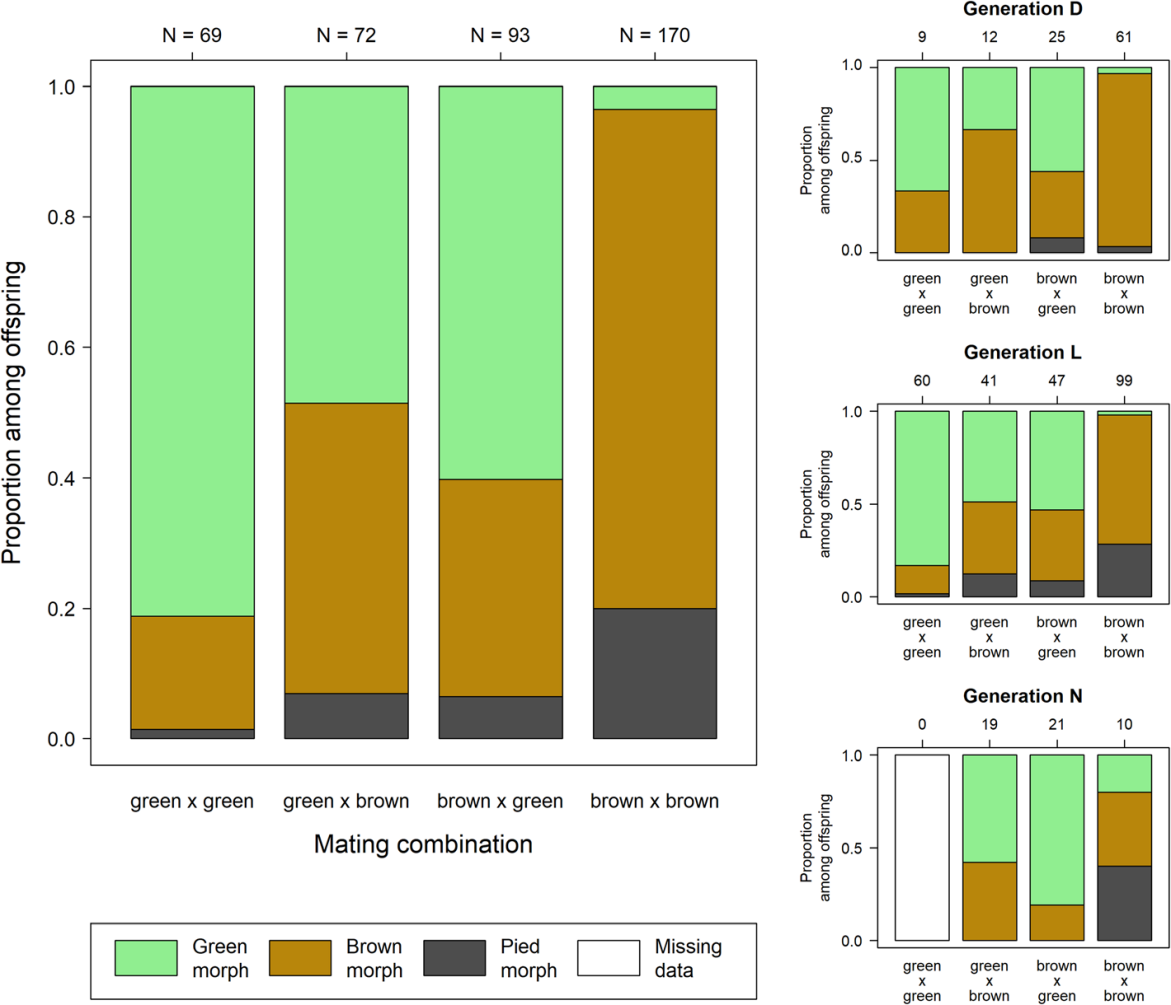
Distribution of offspring morphs across mating combinations separating brown sensu *lato* (i.e. brown and pied combined) from green morphs. Sample sizes in terms of the number of offspring scored are shown on top of the plot

We scored color morphs in 404 offspring from 89 mating pairs (24 green-green, 40 brown-green, 26 green-brown, 48 brown-brown, 9 pied-pied, 5 brown-pied, 1 pied-brown and 1 green-pied) in three cohorts that we call cohorts D, L and N. Since pied individuals are non-green, we refer to brown and pied together as brown sensu *lato* (Fig. 1). Overall, 153 offspring were scored as green, 205 as brown and 46 as pied. The distribution of color morphs across families was very uneven and correlated strongly with the color morphs of the parents (GLMM, χ^2^_7_ = 127.4, *p* < 10^− 15^, Fig. 2). There were several striking patterns: (1) Nearly all offspring from brown-brown and pied-pied matings were brown or pied and not green. (2) In the vast majority, but clearly not all cases, offspring from green-green matings were green. (3) The proportion of green offspring was intermediate in mixed matings and did not depend on whether the female or the male was brown (GLMM, b = − 0.44 ± 0.33, t = − 1.32, *p* = 0.19). (4) Both brown-brown and pied-pied matings produced both brown and pied offspring, but the ratios were approximately inverted with many more offspring matching the parental morph.

In only five cases (3%) we found a green individual in a family of brown-brown matings (sensu *lato*). In cohort D, this applied to a single offspring each in a brown-brown and a brown-pied family with 3 and 2 total offspring, respectively. In cohort N this applied to two single green offspring from separate egg pods of a single brown-brown mating. In cohort L this applied to two out of four offspring from a brown-brown mating. These cases illustrate the occasional appearance of green offspring in brown-brown families.

Overall, these results demonstrate genetic control of both color variants. Besides five cases of mismatches (possible phenocopies, sensu [6]), the simplest explanation for the occurrence of green morphs is a one-locus, two-alleles model with the green allele being dominant over the non-green allele and the locus being located on the autosomes. There are five patterns that allow estimating the frequency of a putative dominant green allele (cohorts L and N only): (P1) The proportion of green individuals from a random sample collected at the field site (35% based on 918 individuals surveyed in the field, [16]), (P2) The overall fraction of brown individuals among green-green mating pairs (17%), (P3) The overall fraction of brown individuals among mixed mating pairs (55%), (P4) The fraction of green-green families that show only green offspring (43%), suggesting that one or both parents were homozygous for the green allele, (P5) The fraction of mixed families that show only green offspring (18%), suggesting that the green parent was homozygous for the green allele.

**Fig. 3:**
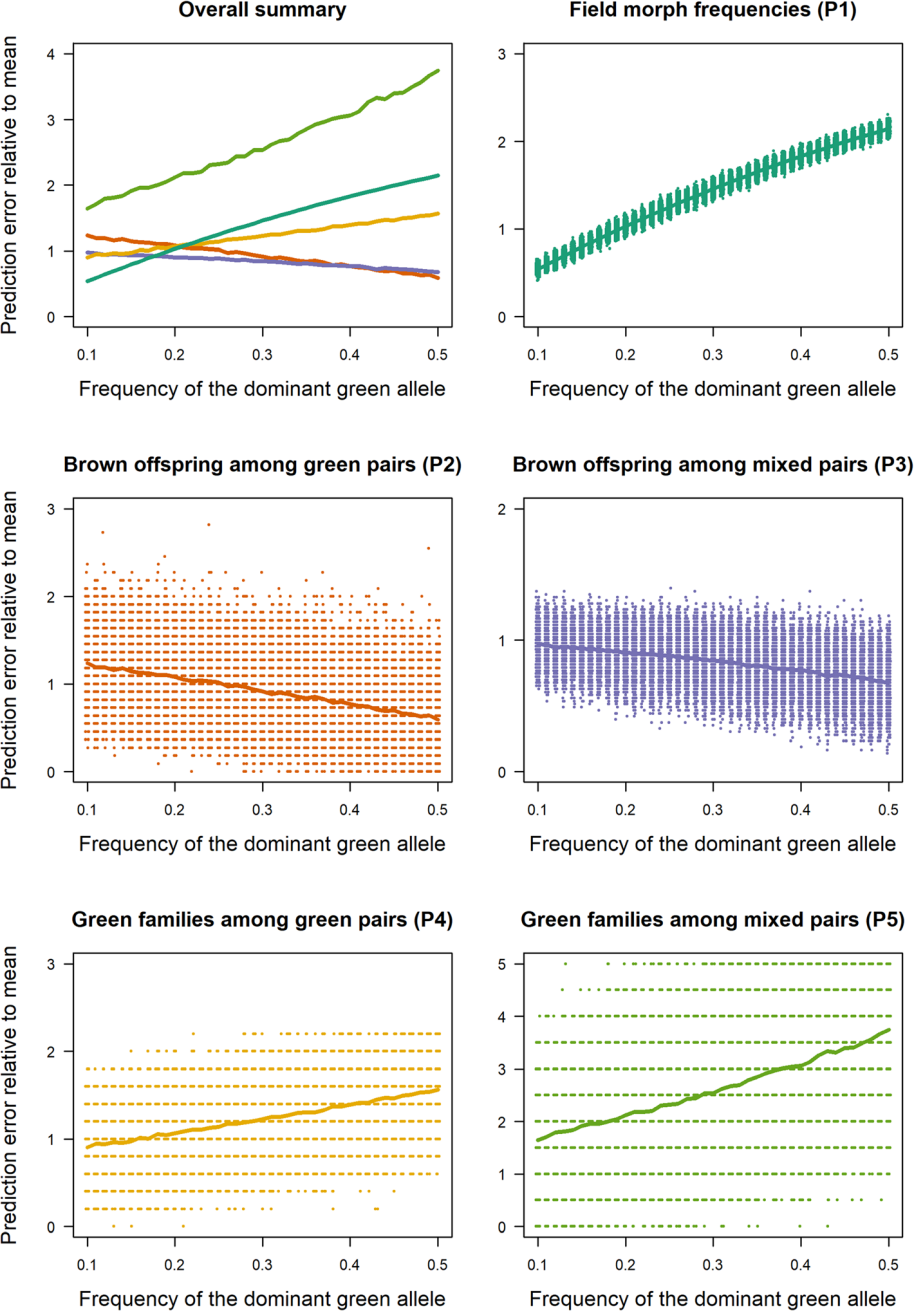
Fit of simulation results across an allele frequency gradient to observed patterns of morph ratios. The x-axis shows the range of allele frequencies of a putative dominant green allele that were simulated. The y-axis shows the mismatch expressed as simulated divided by observed values and values of 1 thus indicate the best match. The upper left plot summarizes results across the five patterns with colors matched to colors used in other subplots

We used a simulation approach across a range of different allele frequencies to estimate the allele frequency that best fits these patterns. It turns out, that all patterns are closely matched with in a one-locus, two-alleles autosomal model when the green allele is dominant and occurs at an allele frequency of *p*_G_ = 0.21 (Fig. 3). Only the fraction of exclusively green families among green-green pairs is not very well matched in our simulation, but this is not surprising, since the total number of occurrences is low and the simulation results are highly variable in this case (Fig. 3).

**Fig. 4:**
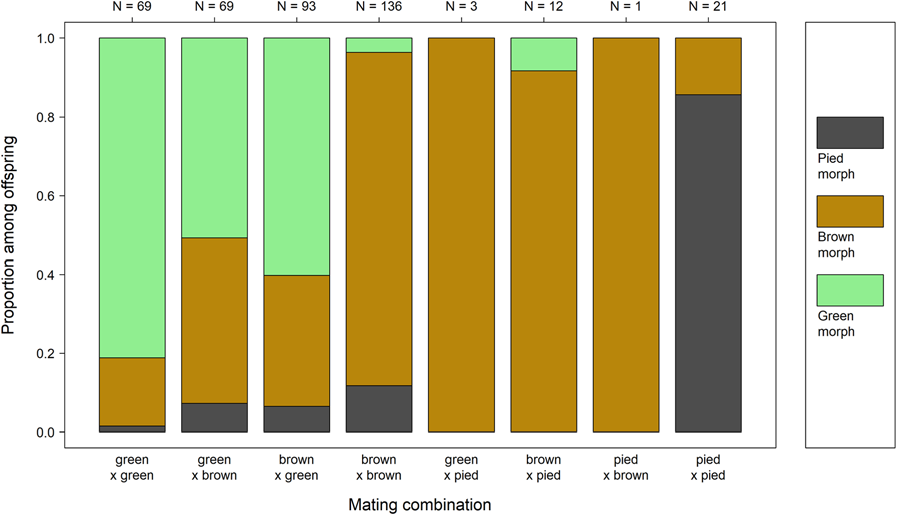
Distribution of offspring morphs across mating combinations separating brown, pied and green morphs. Sample sizes in terms of the number of offspring scored are shown on top of the plot

Sample sizes were lower for pied-pied mating combinations and the pied morph is more difficult to score, so that there were likely more misassignments than between green and non-green morphs. Despite these limitations, the data show that the pied morph is also highly heritable (Fig. 4). Considering low sample size and the possibility of misscoring in fathers or offspring (see discussion and methods section), we refrained from simulating allele frequencies. However, the ratios of offspring morphs seemed so clearly dependent on parental morphs, that simple genetic inheritance is at least possible.

## Discussion

We here report on the inheritance of two color polymorphisms in the club-legged grasshopper *Gomphocerus sibiricus*. The data suggest that the inheritance of a striking green-brown polymorphism that is phylogenetically and geographically widespread among orthopterans, is likely inherited by one autosomal locus with a dominant green allele in the club-legged grasshopper. The best fit to our observations is achieved by a local allele frequencies of the putative green allele of *p*_G_ = 0.21. There are a few cases of green offspring from brown x brown (sensu *lato*) matings that are inconsistent with a one-locus model. These cases might represent the action of genetic modifiers, mixed penetrance of multiple “brown” alleles or environmentally induced phenocopies. A second polymorphism within the non-green category, the pied-brown polymorphism that is rather widespread among species of gomphocerine grasshoppers, is also genetically inherited and it seems possible that the genetic architecture is also simple.

The green color of orthopterans is thought to be produced by biliverdin [12] that is deposited in epidermal cells [30]. Biliverdin is a product of the heme oxygenase pathway (KEGG database, http://www.genome.jp/kegg/) and a number of candidate genes have been identified in the species’ transcriptome [31]. Biosynthesis is likely happening in the hemolymph [30], although this has not formally been shown for the club-legged grasshopper. We predict that the biosynthetic pathway is interrupted in non-green individuals, such that no biliverdin is produced or it is not transported to and deposited in the epidermis. The apparent dominance of the putative green allele, in particular, is consistent with the interpretation of a biosynthetic pathway being switched on/off. Under this assumption, a single functional green allele can be sufficient to produce the pigment, leading to the phenotypic dominance of the green color.

The occurrence of apparent phenocopies that do not match the overall pattern of simple genetic inheritance suggests that the effect of an apparent major gene is affected by genetic modifiers or by the environment. The two mechanisms are not mutually exclusive such that even the few cases in our sample might have different underlying causes. Genetic modifiers might segregate in the population and might act additively or epistatically. Similarly, environmentally induced phenocopies might have affected parents and/and offspring. It seems possible that some green individuals have facultative inactivated the green pigmentation pathway. The environmental triggers of such inactivation are currently unknown and seem to be rare and not among the obvious candidates (temperature, humidity, background color, crowding) reported in other studies [29]. It might be that one of the parents had a functional green allele that was not expressed for unknown reasons. Given that the overall pattern strongly suggests dominant gene action of an autosomal green allele, we consider it less likely that individuals without a functional green copy are able to facultatively express green pigments. In any case, the overall remarkably clear results suggest that a facultative switch-off of green pigmentation is rare in the species.

The identification of the genetic basis of the green-brown polymorphism in an gomphocerine grasshoppers is of significance, because the phenotypic green-brown polymorphism is shared among so many species of orthopterans and polyneopterans (see introduction). The underlying mechanism may or may not be shared across species. Recent data from the green-brown polymorphic genus *Timema* of stick insects shows a pronounced genetic signal on a single scaffold [11, 32]. However, the actual causal gene has yet to be identified. Data from other orthopterans also suggest a strong heritable basis of green-brown polymorphisms in the gomphocerine grasshoppers *Pseudochorthippus parallelus* [28, 33] and *Chorthippus brunneus* [27]. Consistent with the results that we report here, experimental studies shows no plasticity in the club-legged grasshopper [29]. Interestingly, species of orthopterans mostly outside the Gomphocerinae have been reported to show environment-dependent developmental plasticity in the green-brown polymorphism [20–24, 34].

The functional relevance of the green vs brown morphs and the selection pressures that maintain the apparently balanced polymorphism are not yet known. However, crypsis and predator avoidance, possibly in combination with thermoregulation and/or mating advantages are possible explanations. There is indeed evidence that brown morphs have a thermoregulatory advantage in the club-legged grasshopper and the meadow grasshopper *Pseudochorthippus parallelus* [19]. The spatial distribution across large parts of the alpine range shows small and large-scale heterogeneity with temporal stability [16]. This pattern is consistent with small-scale local adaptation in migration-selection balance, although other explanations are possible [16].

The pied-brown polymorphism also shows a genetic basis that is apparently genetically rather simple. Firm conclusions about the exact pattern of inheritance are currently difficult, partly because sample sizes are lower for pied morphs, but also because pied individuals are more difficult to identify, in particular at early developmental stages (more assignments changed brown to pied than the other way around). The mode of inheritance might therefore be simpler than the data might suggest. While early developmental stages are difficult to assign to pied or brown morphs, this also applies to adult males. Last instars of both sexes are easily recognized as pieds, but the distinctness is lost in adult males within a few days after final ecdysis (own observations). In particular, the characteristic black face mask that is typically very prominent in adult females and nymphae of both sexes is usually lost in males with final ecdysis, and the characteristic transverse black-and-white pattern on the sides of the head and pronotum is often blurred or lost due to overall darkening. We had found slight, but significant inter-annual variation in the occurrence of pied morphs (but not of green morphs) in the field [16]. These two observations suggest that unlike the green-brown polymorphism, the pied-brown polymorphism might be phenotypically plastic to some degree.

The pied pattern is likely produced by the distribution of melanin within the head and thorax. Melanin is apparently present in all individuals, thus the causal genetic pathways is less likely to involve the biosynthesis of the pigment itself, but rather the distribution and incorporation in to the integument. The pied pattern shows the characteristics of a disruptive, contour-dissolving pattern [35, 36]. This can make it more difficult for some natural enemies to target their prey. Parasitic flies, for example, seem to be rather common enemies, since we regularly found fly larvae in individuals imported from the field (own observations). The black face mask of pied morphs also produces a disruptive pattern, but may also be involved in mating displays and/or antagonistic interactions, since the frons is presented during males-female and male-male encounters. Involvement in courtship has not yet been studied.

## Conclusions

Our results show remarkably clear and simple inheritance of color morphs in a species of gomphocerine grasshoppers. The results promise potential for future genetic mapping, since loci with a strong effect such as for the green-brown polymorphism in our species should be easy to detect [11, 37]. Unfortunately, the genome of the club-legged grasshopper is very large (approx. 8.8 Gb, [38, 39]), making it difficult to develop genetic resources. We have recently assembled the transcriptome for this species as a basis for differential expression analyses [31]. It will thus hopefully be possible to identify the major gene by RNA sequencing followed by functional verification. Furthermore, the occurrence of possible phenocopies make this a potentially interesting system for studying developmental modifiers.

## Methods

### Source populations

Mating experiments and offspring scoring were conducted in three cohorts. For each cohort, pairs were set up in one year and offspring were scored in the following year(s). Cohort D was formed from field-caught individuals sampled in 2012 and raised in 2013, cohort L was formed from field-caught individuals sampled in 2016 and raised in 2017, cohort N was formed from the same field-caught individuals sampled in 2016 but that were raised in 2018.

Cohort D was founded by parents sampled at a single location in the Swiss Alps in July 2012 (Crans Montana, Valais). Cohorts L and N were founded by parents sampled at two locations in the High Tauern mountain range in the Austrian Alps (Albitzen/Heiligenblut, Carinthia, and Peischlachalm/Karls am Großglockner, East Tyrol) in July/August 2016. While the Austrian and Swiss sites are separated by about 400 km, the mechanistic underpinnings of the green-brown polymorphism are likely to be similar, although allele frequencies might differ. Notably, sequence divergence between Alpine and Chinese populations were shown to be low (1.6% mitrochondrial divergence [31]) making it unlikely that genet differentiation within the Alps is high.

In all cases, we sampled last and second-last instar nymphae (nymphal stages 3 and 4) to ensure virginity. Subjects were raised into the imaginal stage in the laboratory. Upon final molt, the sexes were separated before they became sexually mature.

### General housing conditions

Subjects were maintained in cages of dimensions 22 × 16 × 16 cm^3^ and had access to ad libitum food (potted bundles of cut grass in vials filled with water and sealed by a cotton plug) and water (water-filled vials with a cotton plug in horizontal position such that the plug was soaked with water). Small pots (diameter 4 cm, height 3 cm) filled with a 1:1 mixture of sand and vermiculite were provided as egg laying substrate to adult females. Sand pots were searched 1–2 times per week for egg pods, solid structures that typically contain up to 12 eggs. Eggs were left at room temperature for at least four weeks while they were regularly sprayed with water to keep them moist. Overwintering happened in petri dishes lined with moist filter paper at low positive temperatures (approximately + 4 °C) in refrigerators. Diapause was ended after 3–8 months (1.5 years for cohort N) by transferring petri dishes to room temperature. Most hatching happened after about 10–14 days.

### Morph classification

Color morphs of the club-legged grasshopper can be classified into green, brown, and pied (Fig. 1). Only green individuals show green color while both brown and pied variants lack any distinct green tones altogether. The green color morph is conspicuous and easily scored from the second or third nymphal stage (out of four nymphal stages in this species). Pied morphs are characterized by a transverse black-and-white pattern on the lateral sides of head and pronotum and usually a marked black frons [16]. Because of the lack of green, we consider pied as a variant of brown and therefore analyze the occurrence of green versus brown/pied (brown sensu *lato*) as well as the occurrence of pied relative to brown morphs. Pied morphs are most easily identified in last instar nymphae and in adult females. The identification of pied morphs is less easy in adult males, because they lack the black frons and their lateral pattern is blurred by overall darkening. Pied morphs are also more difficult to score as young nymphae (before the last nymphal stage). Therefore, misscoring is more likely in pied vs. brown than in pied/brown vs. green morphs. Scoring of offspring was done blind to morph mating combination.

### Mating design

We set up all pairwise combinations of green and brown individuals. For cohort L we did so in approximately equal numbers for all mating combinations. As pied individuals occur in much lower frequencies compared to brown and green ones [16], it was not possible to create a full-factorial mating design with sufficient sample size. We therefore created mostly pied x pied mating combinations as to compare them to brown x brown families (here and throughout the text we name the color morph of the female first). Mating pairs were kept separately in cages. In cases were no copulation was observed and no egg pods were laid, males were replaced by another male of the same color morph.

### Offspring rearing and scoring

Hatchlings were transferred from petri dishes to rearing cages on the day of hatching. Hatchlings from different egg pods were kept in separated cages while hatchlings from the same egg pod were housed together. First instar hatchlings are pale at birth, but turn dark within a few hours with no visible morph differences. Second instars (a stage usually reached after about one week) are still mostly dark and sometimes difficult to score. From the third instar onwards (reached about one week after the second instar) it is straightforward to distinguish green from brown individuals and it is usually possible to recognize pied individuals by their black face mask and characteristic lateral pattern.

In cohorts D and N we scored adult individual (cohort D) or last instar nymphae (cohort N) before they were used for other purposes. In cohort L we aimed to acquire color morph phenotypes as early as possible. We therefore decided to score color morphs identities every 2–3 days (which was done by different observers) from the third instar onwards. Each family was scored 3.9 ± 1.9 times (mean ± SD). Since individuals were kept in families and since it is unfeasible to mark subjects individually across their different nymphal stages, morph data is resolved to the level of egg pods. Since we scored the number of offspring family-wise, we were able to identify misscoring that led to an increase in one morph category and a simultaneous decrease in another. In a total of 15 cases (3% of the total), there was a mis-scoring that involves green to brown (9 cases) or brown-to-green (6 cases) scores, which affected 9 out of 53 (17%) of all families. In a total of 16 cases (6% of the ones that included brown/pied), there was a misscoring that involves brown to pied (12 cases) or pied-to-brown (4 cases) changes, which affected 11 out of 53 (21%) of all families. At least with respect to the green morphs, changes are more likely to involve actual miscounting or misscoring rather than changes in color, since previous data based on carefully following single individuals did not show any green-brown or brown-green switch [29]) and later counts in our data usually matched very well with counts prior to the putative misrecording. We resolved ambiguous cases by taking the numbers that were scored most often.

### Statistical analysis

We analysed the effect of mating combination using generalized linear mixed models (GLMM, package lme4 version 1.1–20, [40], in R 3.6.0, [41]) with logit link and binomial error distribution. Cohort and cross-type were fitted as fixed effects and mating pair identity and an observation-level identifier as random effects, the latter controlling for overdispersion. Likelihood ratio tests were used for hypothesis testing. We used the package RColorBrewer version 1.1–2 [42]) for display in one of the figures.

### Simulation

We used a simulation approach to assess if the observed patterns of inheritance match a monogenic inheritance of a dominant green allele. To this end we sampled simulated parental alleles at random from specified allele frequencies. One copy of each parental allele was then randomly inherited to each offspring. The simulation followed closely the sampling design in terms of parental phenotypes as well as samples size of parents and offspring. We then assessed each pattern (see results section) in the offspring generation. The simulation was conducted across a range of allele frequencies (0.1 to 0.9 at steps of 0.01) with 1000 replicates per allele frequency. The distribution across all replicated simulations were compared to the empirically observed patterns. We calculated the ratio of simulated to observed values as a measure of agreement, with values of unity indicating a perfect match. Since allele frequencies may differ between sites, the simulation involved only cohort L and N that were sampled in Austria and for which accurate field morph frequencies were available.

## Supplementary information

**Additional file 1.** Raw data on offspring color morphs from individual egg pods and parental mating combinations.

## Data Availability

All data generated or analyzed during this study are included in the supplementary information files.

## Abbreviations

GLMM: Generalized linear mixed-effects model
KEGG: Kyoto Encyclopedia of Genes and Genomes
Mya: Million years ago
